# Sodium Thiosulfate in the Pregnant Dahl Salt-Sensitive Rat, a Model of Preeclampsia

**DOI:** 10.3390/biom10020302

**Published:** 2020-02-14

**Authors:** Fieke Terstappen, Sinéad M. Clarke, Jaap A. Joles, Courtney A Ross, Michael R. Garrett, Magdalena Minnion, Martin Feelisch, Harry van Goor, Jennifer M. Sasser, A. Titia Lely

**Affiliations:** 1 Department of Obstetrics, Wilhelmina Children’s Hospital, University Medical Center Utrecht, 3508GA Utrecht, The Netherlands; sineadclarke92@gmail.com (S.M.C.); a.t.lely@umcutrecht.nl (A.T.L.); 2 Department of Developmental Origins of Disease (DDOD), Wilhelmina Children’s Hospital, University Medical Center Utrecht, 3508GA Utrecht, The Netherlands; 3 Department of Nephrology and Hypertension, University Medical Center Utrecht, 3508GA Utrecht, The Netherlands; J.A.Joles@umcutrecht.nl; 4 Department of Pharmacology and Toxicology, University of Mississippi Medical Center, Jackson, MS 39216-4505, USA; cross3@umc.edu (C.A.R.); MRGarrett@umc.edu (M.R.G.); jsasser@umc.edu (J.M.S.); 5 Clinical and Experimental Sciences, Faculty of Medicine, University of Southampton, Southampton SO16 6YD, UK; M.Minnion@soton.ac.uk (M.M.); M.Feelisch@soton.ac.uk (M.F.); 6 NIHR Southampton Biomedical Research Center, Southampton General Hospital, Southampton SO16 6YD, UK; 7 Department of Pathology and Medical Biology, University Medical Center Groningen, University of Groningen, 9713GZ Groningen, The Netherlands; h.van.goor@umcg.nl

**Keywords:** blood pressure, cardiovascular, Dahl salt-sensitive rats, fetal growth restriction, hydrogen sulfide, preeclampsia, sodium thiosulfate, therapeutics

## Abstract

Aberrant production of hydrogen sulfide (H_2_S) has been linked to preeclampsia. We hypothesized that sodium thiosulfate (STS), a H_2_S donor, reduces hypertension and proteinuria, and diminishes fetal growth restriction in the Dahl salt-sensitive (S) rat, a spontaneous model of superimposed preeclampsia. In addition to a control group (n = 13), two groups received STS via drinking water at a dose of 2 g (n = 9) or 3 g per kg body weight per day (n = 8) from gestational day (GD) 10 to 20. Uterine artery resistance index was measured (GD18), urinary protein excretion rate was determined (GD19), and blood pressure and fetal outcomes were evaluated (GD20). At 2 g, STS had no effect on preeclamptic symptoms or fetal outcome. At 3 g, STS reduced maternal hypertension (121.8 ± 3.0 vs. 136.3 ± 2.9), but increased proteinuria (89 ± 15 vs. 56 ± 5 mg/24 h), and relative kidney weight (0.86 ± 0.04 vs. 0.73 ± 0.02%). Fetal/placental weight ratio was reduced (3.83 ± 0.07 vs. 4.31 ± 0.08) without affecting litter size. No differences in uterine artery flow or renal histological damage were noted across treatment groups. While these data suggest a promising antihypertensive effect that could imply prolongation of preeclamptic pregnancies, the unfavorable effects on proteinuria, kidney weight, and fetal/placental weight ratio implies that clinical implementation of STS is contra-indicated until safety for mother and child can be verified.

## 1. Introduction

Preeclampsia (PE) complicates 3% to 5% of all pregnancies and is a leading cause of maternal and perinatal mortality [1,2]. PE presents after 20 weeks of gestation and is characterized by hypertension accompanied by maternal organ disturbances (including proteinuria), as well as fetal growth restriction (FGR) [3]. Iatrogenic premature delivery is often required to preserve maternal health and to prevent stillbirths. Because PE is directly life-threatening to mother and child and leaves an unmitigated long-term cardiovascular risk for both in its wake, there is a considerable unmet need for treatment options [2,3,4,5,6].

The complex pathogenesis of PE is not fully understood. PE is considered a sequel to an undesirable interaction between placental and maternal constitutional factors [2,5]. The hypoxic placenta secretes proinflammatory cytokines (tumor necrosis factor alpha [TNF-α] and interleukin-6) and anti-angiogenic factors (soluble Fms-like tyrosine kinase 1 [sFlt1] and soluble endoglin [sENG]) [2,7]. The resulting systemic endothelial dysfunction eventually leads to the cardinal symptoms of PE [2,5].

Dysregulation of the hydrogen sulfide (H_2_S) pathway has been suggested to contribute to the underlying mechanisms of PE and FGR. H_2_S contains pro-angiogenic, anti-inflammatory, vasodilatory, and antioxidant properties, and thus is important in vascular adaptation [8,9,10]. This gasotransmitter is endogenously produced in the placenta from L-cysteine by enzymes including cystathionine β-synthase (CBS) and cystathionine γ-lyase (CSE) [11]. Several studies have shown that placental production of H_2_S, expression of CBS and CSE, and plasma H_2_S concentrations are all reduced in pregnancies complicated by PE and FGR [8,10]. These aberrant H_2_S levels have been linked to the anti-angiogenic environment and endothelial dysfunction associated with PE [12,13].

The potential of H_2_S as a mechanism-based therapy for PE has been tested with several H_2_S donors [11,14]. In rat models of PE, administration of sodium hydrosulfide (NaHS) and GYY4137 (synthetic H_2_S donor) reduced hypertension and circulating sFlt-1 levels, and increased fetal weight [8,15]. More recently, sodium thiosulfate (STS, Na_2_S_2_O_3_) caught clinical interest as this H_2_S donor, in contrast to other H_2_S donors, is already approved for human use (e.g., in calciphylaxis, cyanide poisoning or protection against cisplatin toxicity) and can conveniently be administered orally [9,16]. Thiosulfate produces H_2_S through enzymatic and non-enzymatic pathways in a glutathione-dependent manner. H_2_S, glutathione disulfide, and labeled sulfite are generated when mitochondria are incubated with glutathione, STS, and oxygen [17]. Indeed, we found that STS ameliorated hypertension and proteinuria in an angiotensin-II model of cardiac disease in male rats [16]. However, STS has not yet been tested in an animal model of PE.

Dahl salt-sensitive (S) rats are a spontaneous model of hypertension and kidney disease [18,19]. Pregnant Dahl S rats develop features of PE, including exacerbation of hypertension and proteinuria, FGR, increased placental hypoxia, and increased plasma levels of sFlt-1 and TNF-α [20]. Pregnant Dahl S rats showed glomerulomegaly during late pregnancy as compared with virgin Dahl S rats, a trend not observed in virgin versus pregnant non-Dahl S rats [20]. As in preeclamptic women, the pregnant Dahl S rat exhibits sustained high uterine artery resistance (UARI) with characteristic notching in Doppler waveforms [20].

In this study, we test whether oral administration of STS in pregnant Dahl S rats attenuates the maternal preeclamptic phenotype (high blood pressure, proteinuria, and high UARI) and improves fetal outcomes (fetal and placental weight, litter size).

## 2. Materials and Methods

### 2.1. Animals

A total of 40 nulliparous Dahl S female rats (12 to 14 weeks, 232 ± 11 g) were acquired from Dr. Garrett’s colony at the University of Mississippi (MS) Medical Center (UMMC), Jackson, MS. In order to achieve pregnancy, one to two female rats were housed with one Dahl S male rat per cage under controlled conditions (12 h light/dark cycle, 23 °C). Presence of sperm on vaginal smears indicated gestational day (GD) one. The rats had free access to water and normal chow (TD7034, 0.3% NaCl, Harlan Teklad, Madison, WI, USA). Conventional (type 3) cages were provided with sani-chip bedding (7090 Teklad Laboratory Grade) and enriched with a paper straw as nesting material. Ten Dahl S rats were excluded from analysis due to failure to achieve pregnancy, failure to accurately determine GD1, or death resulting from post-operative complications. As a result, 30 rats were included in the final analysis. The experiments were carried out in accordance with the National Institutes of Health Guide for the Care and Use of Laboratory Animals and approved by the UMMC Institutional Animal Care and Use Committee (protocol 1344A “New animal models to investigate hypertensive complications of pregnancy”, approved on 24 September 2015, amended on 6 November 2017, and renewed as protocol 1344B on 1 June 2018), and reported according to ARRIVE guidelines.

### 2.2. Study Protocol

#### 2.2.1. Sodium Thiosulfate Treatment

**Figure A1:**
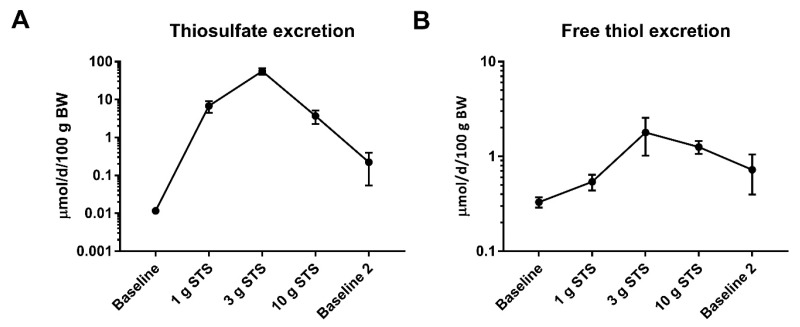
**Mean urinary thiosulfate excretion per dose.** In a pilot study, sodium thiosulfate (STS) was orally administered via the drinking water at incremental doses to four healthy female Sprague Dawley rats. Highest urinary excretions of (**A**) thiosulfate and (**B**) free thiol were reached at a dose of 3 g/kg/day STS. BW, body weight. Means ± SEM.

Dahl S rats were stratified into control (n = 13) and treatment groups. Littermates were assigned to different treatment groups, and we checked that average baseline urinary protein excretion rates (one week prior to pregnancy) were similar among the three groups (a cardinal feature of PE) to ensure indirectly that all groups had equal degrees of pre-existent renal injury. STS pentahydrate (EMSURE, Inc, Sigma) was administered at two different doses, 2 g per kg body weight per day (n = 9) and 3 g per kg body weight per day (n = 8), to two different subsets of rats via drinking water in their cage from GD10 through to GD20. Drinking water containing STS was prepared based on weight gain over the previous three days and based on each rat’s average daily water consumption over the previous three days. In other words, every three days, the STS concentration in drinking bottles was reformulated to account for gestational weight gain and possible changes in water intake throughout pregnancy to ensure that each individual rat was ingesting the appropriate dose. Due to this administration route, shared housing was no longer possible after the start of the treatment. The chosen dose was based on a pilot study performed in female rats (Figure A1 in Appendix A).

#### 2.2.2. Renal Injury Measurements

The rats were placed in metabolic cages for 24 h urine collection on two occasions, prior to mating (baseline) and at end of pregnancy (from GD19 to 20). Sodium azide (Fisher Chemical, Inc, Waltham, MA, USA) was added to the urine collection container to prevent bacterial growth. Urinary protein concentration was measured using the Bradford Assay (BioRad Laboratories, Hercules, CA, USA), and proteinuria was defined as >20 mg/24 h. Urea was measured colorimetrically (DiaSys Diagnostic Systems, GmbH, Holzheim, Germany) in plasma obtained on GD20.

#### 2.2.3. Uterine Artery Resistance Index

Uterine artery power Doppler velocimetry measurements were performed under isoflurane anesthesia/1LO_2_ (NDC11695-6776-2, Henry Schein Animal Health, Dublin, OH, USA; induction with 3% and maintenance with 1.5% to 2%**)** in all rats with a Vevo 3100 (FUJIFILM VisualSonics, Inc, Toronto, ON, Canada) using a 30 Hz transducer on GD18. The Doppler waveform of the uterine artery was recorded. The peak systolic flow velocity (PSV) and end-diastolic flow velocity (EDV) values of three waves per location were determined to calculate the uterine artery resistance index (UARI) as follows: UARI = (PSV-EDV)/PSV. UARI was determined bilaterally at two to three different locations along the uterine artery, from which mean UARI was calculated.

#### 2.2.4. Terminal Mean Arterial Pressure (MAP) Measured under Anesthesia

At the time of tissue harvesting, rats were anesthetized using isoflurane (induction with 3% and maintenance with 2%). The terminal MAP (primary outcome) in all rats in the three groups (control and two different STS dosages) was measured via the abdominal aorta. MAP measurements were recorded using the PowerLab system with the LabChart software (ADInstruments, Sydney, Australia), and a MLT0699 Disposable BP Transducer (ADinstruments) that was calibrated daily. The use of anesthetized terminal blood pressure was considered a valid method for assessing hypertension in rats [21]. Accompanying heart rate was determined.

#### 2.2.5. Tissue and Blood Collection

Tissue collection was performed on GD20. A terminal blood sample was extracted from the abdominal aorta, after which the organs were perfused blood free with phosphate-buffered saline. The number of viable and resorbed fetuses in each uterine horn was noted. Subsequently, placental weight, fetal weight, and crown-rump length for each viable fetus was recorded and averaged per litter. Fetal/placental weight ratio was calculated for each individual unit before averaging per litter. Kidneys, hearts, and spleens were blotted dry on filter paper and, then, weighed. Hematocrit was determined using glass micro-hematocrit heparinized capillary tubes and Sorvall Legend Micro 17 microcentrifuge (Fisher Scientific, Inc, Waltham, MA, USA).

#### 2.2.6. Plasma and Urinary Thiosulfate and Sulfate Concentration

The concentrations of thiosulfate (S_2_O_3_^2−^) and sulfate (SO_4_^2−^) in the 24 h urine collected at the end of pregnancy (GD19 and GD20) and in plasma collected on GD20 were determined by ion chromatography with mass spectrometry detection using a reagent-free high-pressure Dionex^TM^ ICS-5000 MSQ system attached to an AS-AP autosampler (Thermo Fisher Scientific, Hemel Hempstead, UK). Thiosulfate and sulfate were separated from other anions present in the samples using a Dionex IonPac AS16 2 × 250 mm analytical column, protected by an AG16 2 × 50 mm guard column, both kept at a constant temperature of 30 °C. An eluent generator with a potassium hydroxide (KOH) cartridge was used to produce the following gradient: 20 mM from 0.1 to 4.0 min, followed by 30 mM until 8.0 min at a constant flow rate of 0.38 mL/min. Total run time was 10 min with retention times of 4.2 min and 6.1 min for sulfate and thiosulfate, respectively. The injection volume was 2.5 µL. The system was equipped with an Anion Electrolytically Regenerated Suppressor (AERS 500e, 2 mm) with an applied current of 29 mA. The quadrupole detector was coupled to the IC via an electrospray ionization interface (ESI) and operated in negative SIM mode at 97 and 113 *m/z* for the monoprotonated anions of sulfate and thiosulfate, respectively. The capillary voltage was maintained at 2.5 kV, while the cone voltages were 70 V and 55 V for sulfate and thiosulfate, respectively. Probe temperature was kept at 500 °C. Prior to analysis, samples were subjected to ultrafiltration using Amicon^©^ Ultra Centrifugal Filters with a 10 kD cut-off to remove proteins. The membrane of these filters was found to retain a considerable portion of sulfate and thiosulfate, in particular in the low concentration range. Therefore, all calibration standards used for quantification were also subjected to ultrafiltration prior to injection to account for analyte loss using this sample processing method. Detector responses were found to be linear up to concentrations of 25 µM with a detection limit of approximately 100 nM (S/N ≥ 3) for either anion. Daily urinary excretion values were calculated from the molar concentrations of either anion taking total volumes of the urines collected into account.

#### 2.2.7. Renal Histology

One longitudinal section of the left kidney was immersed in 10% buffered formalin for 48 h, after which it was immersed in 70% ethanol until embedment in paraffin. The paraffin-embedded kidney sections were cut into 4 μm sections and stained with periodic acid staining (PAS). Focal glomerulosclerosis (FGS) and interstitial fibrosis were scored by a blinded investigator. FGS lesions were scored positive by the presence of focal and segmental glomerular scarring and also obliteration of the glomerular capillaries with enhanced mesangial matrix expansion and adhesion formation between the tuft and the capsule of Bowman. The severity of FGS was scored in a semiquantitative manner on a scale of 0 to 4+ in PAS stained sections. Fifty glomeruli were scored and the average per animal was expressed as percentages. Thus, the maximum score for FGS lesions was 400% [22]. Interstitial fibrosis was scored by the presence of significant interstitial broadening of the peritubular area combined with loss of the integrity of the tubular basement membrane. Kidneys were scored for the percentage of involvement as follows: 0 to 5%, 5% to 10%, 10% to 25%, 25% to 50%, and 50% to 100%. Mean glomerular area was determined in Aperio ImageScope (v12.1.0.5029, Leica Biosystems, Dublin, Ireland) by encircling the capsule of Bowman of 50 glomeruli per kidney. The urinary space was calculated by subtracting the glomerular tuft area from the individual glomerular area. We lost one kidney histology sample from a dam treated with STS at 3 g per kg body weight per day.

#### 2.2.8. Placental Histology

One placenta per rat was randomly selected for paraffin embedding (similar procedure as for the kidneys). Placentas were cut transverselyin, the center as 4 μm sections, and stained with hematoxylin and eosin (HE). The area of labyrinth and basal zone were visually determined as the first layer mostly consists of trophoblasts and the second layer of spongiotrophoblasts [23]. The investigator was blinded to the groups. Due to cutting artifacts, determination of the layers was not possible in one placenta of the control group and one placenta from the 2 g STS group.

#### 2.2.9. Statistical Analyses

All data were analyzed using GraphPad Prism 8.0.1 (GraphPad Software, Inc., San Diego, CA, USA). Data were determined to be parametric or non-parametric using Shapiro–Wilk. Statistical analyses were performed using one-way ANOVA with Dunnett’s multiple comparison (parametric data) or Kruskal–Wallis test (non-parametric data). The thiosulfate and sulfate concentrations were tested with Brown–Forsythe and Welch ANOVA with Dunnett’s multiple comparison. Data were presented as mean ± S.E.M unless stated otherwise. A two-sided p-value below 0.05 was considered significant. The experimental unit was an individual pregnant dam. A priori power calculation was based on that a MAP difference of 5 mmHg (primary outcome) would be of clinical interest (effect size f = 2.5), with a power of 0.95, alpha of 0.05, three groups resulted total sample size of 9 (G*Power 3.1.9, Düsseldorf, Germany).

## 3. Results

### 3.1. Effect of STS Treatment on Renal Function Parameters

Our lab previously showed that proteinuria in healthy Sprague-Dawley rat is 12 ± 3 mg/24 h during late pregnancy [20]. Treatment with 2 g STS per kg body weight per day had no effect on proteinuria on GD19 nor on plasma urea on GD20 (Table 1). Treatment with 3 g STS per kg body weight per day resulted in significantly higher proteinuria on GD19 as compared with controls (89 ± 15 mg/24 h vs. 56 ± 5 mg/24 h, *p* = 0.03). Water consumption and urine production (ml/kg/hour) were not significantly different between the treatment groups (Table 1).

### 3.2. Effect of STS Treatment on Uterine Artery Resistance Index

**Figure 1:**
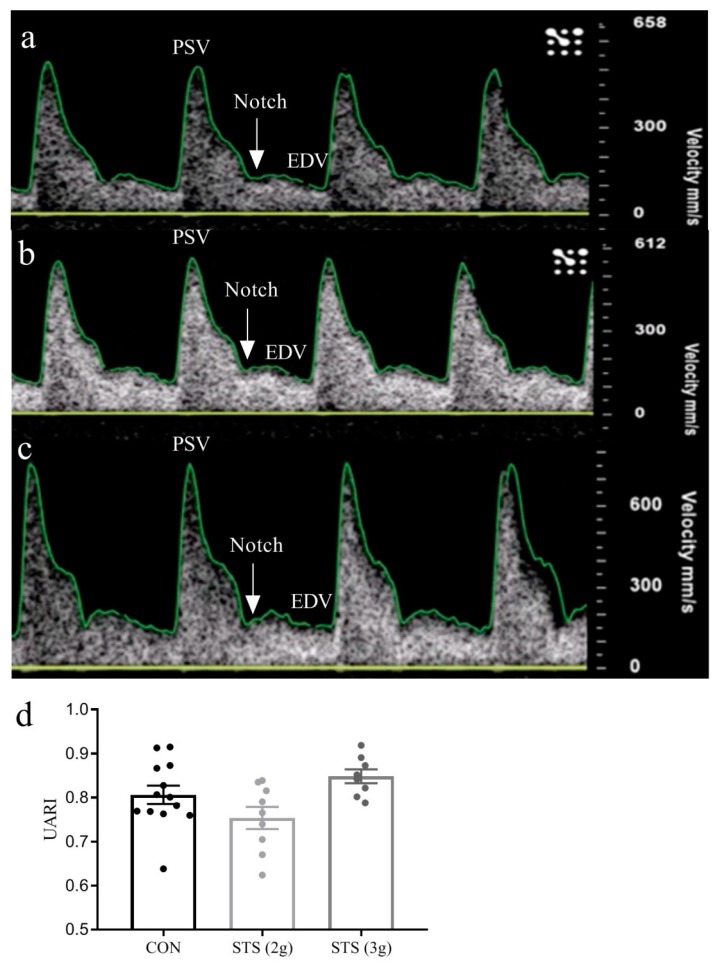
Uterine artery resistance index on GD18. Examples of Doppler waveforms in a (**a**) control dam; (**b**) dam treated with 2 g STS per kg body weight per day; and (**c**) dam treated with 3 g STS per kg body weight per day, white arrows indicate notching; (**d**) treatment with 2 g STS (n = 9) and 3 g STS (n = 8) showed no significant difference in UARI as compared with the control (n = 13) with persistence of characteristic notching tested with one-way ANOVA Dunnett’s multiple comparisons test, data presented as mean ± S.E.M. CON, control; EDV, end-diastolic flow velocity; GD, gestational day; PSV, peak systolic flow velocity; STS, sodium thiosulfate; UARI, uterine artery resistance index; 2 g, 2 g per kg body weight per day; and 3 g, 3 g per kg body weight per day.

The UARI in healthy Sprague-Dawley rats is 0.55 ± 0.02 at the end of pregnancy [20]. In this study we, again, observed elevated UARI with characteristic notching in the ultrasound waveforms in the Dahl S dams (Figure 1a–c). Although there was a treatment effect across the groups (*p* = 0.03), UARI was not different when treatments with 2 g and 3 g STS per kg body weight per day were compared with the control (respectively, *p* = 0.15 and *p* = 0.31, Figure 1d).

### 3.3. Effect of STS Treatment on MAP

**Figure 2:**
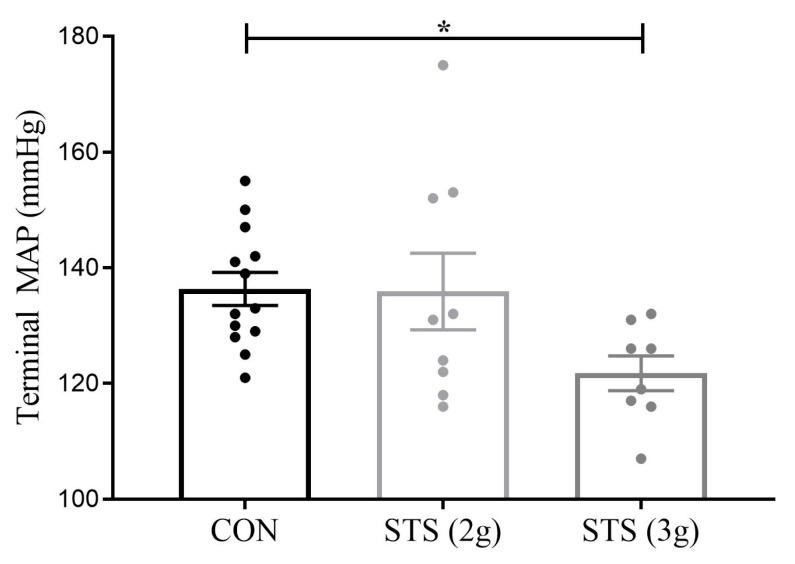
Mean arterial pressure in the Dahl S rat treated with STS versus control. MAP was not different between 2 g STS per kg body weight per day (n = 9) and the control (n = 13) but was significantly reduced by 3 g STS per kg body weight per day (n = 8) as compared with the control dams tested with one-way ANOVA Dunnett’s multiple comparisons test. MAP, mean arterial pressure; CON, control; STS, sodium thiosulfate; 2 g, 2 g per kg body weight per day; and 3 g, 3 g per kg body weight per day. Data presented as mean ± S.E.M. * *p* < 0.05.

During late pregnancy, Sprague-Dawley rats had a MAP of 97 ± 6 mmHg [24] with a heart rate of 326 ± 15 [unpublished data] when measured under anesthesia. Terminal MAP (under anesthesia) was not different between pregnant Dahl rats treated with 2 g STS per kg body weight per day and the control (135.9 ± 6.6 vs. 136.3 ± 2.9 mmHg, *p* = 0.99, Figure 2). In contrast, Dahl S rats treated with 3 g STS per kg body weight per day, demonstrated a significant decrease in terminal MAP as compared with the control group (121.8 ± 3.0 vs. 136.3 ± 2.9 mmHg, *p* < 0.05, Figure 2). Heart rate was also decreased in a dose-dependent manner following STS treatment, CON (n = 10) 359 ± 7 bpm versus STS 2 g (n = 7) 333 ± 9 bpm versus STS 3 g (n = 8) 321 ± 8 bpm (*p* = 0.006, Dunnett’s multiple comparison CON vs. STS 2 g *p* = 0.07 and CON vs. 8 STS 3 g *p* = 0.004).

### 3.4. Effect of STS Treatment on Fetal and Placental Outcomes

Our lab previously showed that fetal weight was 2.46 ± 0.17 g, crown-rump length 3.20 ± 0.09 cm, viable litter size of 13.7 ± 0.58, fetal resorption of 0.47% ± 046%, placental weight 0.56 ± 0.01 g, and fetal/placental weight ratio of 4.49 ± 0.30 in healthy Sprague-Dawley rats on GD20 [20]. Dahls S rats showed lower fetal weight, length, litter size, and placental weight, and higher fetal resorption and fetal/placental weight ratio (Table 2). STS treatment did not affect fetal weight, fetal crown-rump length, litter size, or fetal demise. In addition, placental weight did not differ among groups. However, treatment with STS caused a decrease in fetal/placental weight ratio in the Dahl S rat administered 3 g per kg body weight per day (3.83 ± 0.07 vs. 4.31 ± 0.08; *p* < 0.01).

### 3.5. Effect of STS Treatment on Gestational Weight Gain and Organ Weight

STS administered at 2 g per kg body weight per day showed no effect on relative renal and heart weight (Table 3). When administered at 3 g per kg body weight per day, STS resulted in a higher relative total kidney weight as compared with the control groups (0.86 ± 0.04% vs. 0.72 ± 0.02%, *p* < 0.01). Gestational weight gain from GD0–19 was lower in the 3 g STS treated group as compared with the control group (Table 1).

### 3.6. Plasma and Urinary Thiosulfate and Sulfate Concentration

**Figure 3:**
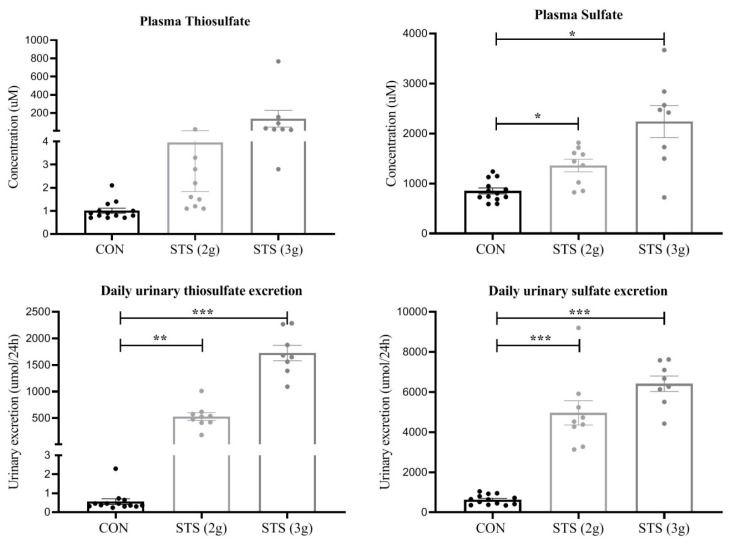
Plasma concentration and daily urinary excretion of thiosulfate and sulfate in the Dahl S rat treated with STS versus a control. Plasma concentrations and urinary excretion between the control dams (n = 13) and treated dams dosed at 2 g STS per kg body weight per day (n = 9) or 3 g STS per kg body weight per day (n = 8) were tested with Brown–Forsythe and Welch ANOVA and Dunnett’s multiple comparison test. CON, control; STS, sodium thiosulfate; 2 g, 2 g per kg body weight per day; and 3 g, 3 g per kg body weight per day. Data presented as mean ± S.E.M. * *p* < 0.01; ** *p* < 0.001; *** *p* < 0.0001.

Plasma concentrations and daily urinary excretion values for thiosulfate and sulfate increased in a dose-dependent manner with STS administration (Figure 3).

When we evaluated the plasma concentrations and urinary excretions levels, independent of the groups, we found that plasma thiosulfate correlated most strongly with MAP (Table 4).

### 3.7. Effect of STS Treatment on Glomerular Injury

**Figure A2:**
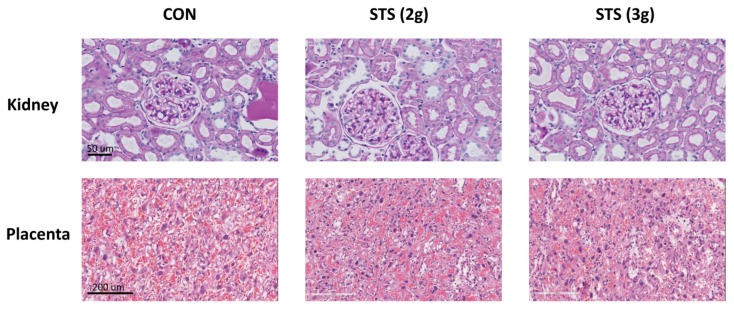
Representative example of glomeruli in PAS staining and labyrinth zone of placentas in HE staining in control and STS treated with 2 g and 3 g in Dahl S rat. CON, control; Dahl S, Dahl Salt-Sensitive rat; STS, Sodium Thiosulfate; 2 g, 2 g per kg body weight per day; 3 g, 3 g per kg body weight per day.

A previous study in our lab showed glomerulomegaly in pregnant Dahl S rats as compared with pregnant Sprague-Dawley rats [20]. Histological evaluation of kidneys collected in our study showed that renal damage caused by pregnancy in the Dahl S rat was neither ameliorated nor exacerbated by 2 g or 3 g STS per kg body weight per day STS (Figure A2 and Table 5). Interstitial fibrosis was <5% in all cases and no vessel abnormalities were observed.

### 3.8. Effect of STS Treatment on Placenta Histomorphology

A previous study reported that the labyrinth zone is 22.1 mm^2^ and the basal zone is 5.0 mm^2^ in Sprague-Dawley rats on GD19.5 [23]. There were no differences in the (proportional) layers across groups (Table 6 and Figure A2).

## 4. Discussion

This study shows that prenatal administration of STS at 2 g per kg body weight per day did not improve or worsen the PE or FGR phenotype in the Dahl S rat. Administration of STS at 3 g per kg body weight per day, however, did reduce maternal blood pressure, but also exacerbated proteinuria, increased renal weight, reduced gestational weight gain, and decreased the fetal/placental weight ratio.

Maternal hypertension is the cardinal feature of PE and the main reason that iatrogenic premature delivery is required. We observed that STS administered at 2 g per kg body weight per day was insufficient to reduce maternal blood pressure. However, when dosed at 3 g per kg body weight per day, STS significantly reduced maternal blood pressure by 14 mmHg. This is clinically interesting because reduced maternal blood pressure would allow the prolongation of preeclamptic pregnancies, which would benefit fetal outcomes. Water intake and urine production, as measured on GD19, and hematocrit as measured on GD20, did not differ between the groups, and as such, the observed reduction in MAP does not appear attributable to hypovolemia. However, heart rate was also dose-dependently reduced in the treated groups versus the control group. As the induction and maintenance of anesthesia was similar, this would seem to suggest increased parasympathetic sensitivity. The inverse correlation between plasma levels and urinary excretion rates of thiosulfate and sulfate and arterial pressure was strongest for plasma thiosulfate, suggesting direct vasodilation. STS has not been tested in a PE setting before, our findings are in line with a previous study that demonstrated STS’s antihypertensive effect in an angiotensin-II male rat model of cardiac disease, and further substantiate a large body of literature that implicates a deficit in H_2_S in the pathophysiology of hypertension [9,16,25,26]. However, N-acetyl-l-cysteine (NAC), the only H_2_S stimulator tested to date in pregnant humans, failed to ameliorate PE in humans [27]. The beneficial effects from our study, therefore, cannot be attributed with certainty to H_2_S production alone, but potentially combined with other effects caused by STS.

Proteinuria is another hallmark feature of PE. Dosed at 2 g per kg body weight per day, STS had no significant effect on gestational proteinuria. However, exacerbated proteinuria and renal enlargement were observed in the dams treated with STS at a dose of 3 g per kg body weight per day. To our knowledge, the detrimental renal effects after treatment with H_2_S have not been described previously, nor has reduced gestational weight after prenatal administration of H_2_S donors been reported. However, in previous studies, STS was administered only once or twice throughout the respective studies and dosed at concentrations ranging from 0.5 to 2.0g per kg body weight in animal studies and up to 25 g in nonpregnant human studies [16,28,29]. In our study, the drug was administered more frequently as compared with these previous studies, and consequently, plasma concentrations and urinary excretions of thiosulfate and sulfate reached very high levels in a dose-dependent manner. Although the higher dose combined with the longer administration period could have resulted in toxic effects in the kidney, especially in the Dahl S rat that is susceptible to renal injury, histological examination of glomerulosclerosis or glomerular area did not show differences between the treated and untreated group.

The presence of FGR as another consequence of placental insufficiency is mimicked by the Dahl S rat, and the fetal weight in our untreated group was comparable with the reduced fetal weight described previously [20,30]. When administered at 3 g per kg body weight per day, STS caused a decrease in fetal/placental weight ratio. This decrease suggests worsening of placental insufficiency; however, this is difficult to conclude in the absence of changes in uterine artery blood flow, fetal weight, placental weight, or area of placental layers. While treating hypertensive disorders of pregnancy could result in prolongation of gestation, antihypertensive drugs, such as labetalol, have been associated with an increased risk of FGR; this detrimental effect is possibly due to a sudden overcorrected blood pressure resulting in (temporarily) decreased uteroplacental blood flow, and therefore impaired fetal growth [31,32]. Effects of STS on fetal outcomes in animal models of healthy pregnancy or PE have not been studied previously.

Thiosulfate uptake occurs in the gastrointestinal tract and, after circulating, is either excreted by the kidneys or oxidized by mitochondria [17,33,34]. In the presence of hydroxyl radicals or peroxides, STS can react with GSSG (oxidized glutathione) resulting in reduced glutathione [17]. Furthermore, STS also has potential to produce hydrogen sulfide by its reaction with trans-sulfuration enzymes, such as thiosulfate sulfur transferase can also convert thiosulfate to H_2_S [33]. We made use of this mitochondrial oxidation pathway to confirm bioavailability after oral application, as we were not able to measure H_2_S as such. Urinary excretion of thiosulfate and sulfate was elevated above baseline in both treatment groups, showing that thiosulfate was absorbed well. Interestingly, plasma thiosulfate concentrations in dams of the 2 g STS group overlapped with those of the control group, while the 3 g STS group showed a much more robust increase. This suggests that the chosen daily STS dose of 2 g per kg body weight was at the border of regulatory (not metabolic) capacity in pregnant Dahl S rats. Plasma sulfate concentrations, however, revealed a clearer dose proportionality, indicating that most thiosulfate was oxidized

Our study had various strengths. This was the first time that the antihypertensive potential of STS was tested in a (complicated) pregnancy setting and the first time that a H2S donor was tested as a potential therapeutic candidate in a spontaneous animal model of PE. In addition, STS’s oral administration route was a highly desirable choice in terms of potential clinical implementation and indeed turned out to be a highly efficient route. We tested two doses of STS. Finally, the effect of a H_2_S donor on UARI was evaluated as a potential mechanism by which H_2_S exerts its effects.

While we ensured adequate intake of STS to the best of our capability by adjusting the concentration of STS in drinking water every three days, we acknowledge the limitation of the oral administration route regarding certainty of the exact dose. However, the very high concentrations of thiosulfate and sulfate measured in plasma and urine documented successful STS intake and efficient metabolic conversion. We also could not compare the bioavailability by oral route versus intraperitoneal injection. We could not determine whether the reduced gestational weight gain in the 3 g STS group was attributed to reduced food intake as this was not measured.

### Future Perspectives

STS administered at 3 g per kg body weight per day appears to reduce maternal MAP, which holds promise for prolongation of preeclamptic pregnancies, and thus fetal outcomes. The H_2_S pathway remains a promising target of research in the search for much needed treatment options for preeclampsia and fetal growth restriction. However, the H_2_S donor, STS, at this dose, appears to worsen placental insufficiency and proteinuria, and to cause kidney enlargement after long-term daily treatment, and thus cannot be recommended for implementation in clinical practice for pregnancy-related diseases at this stage. Further tests are needed to shed light on how STS influences healthy pregnancies and pregnancies complicated by placental insufficiency, and whether the duration of treatment plays a role regarding potential detrimental outcomes. To gain conclusive insight and understand of the mechanisms of action underlying our detrimental findings, future studies should apply STS in other models of PE to determine whether STS can be beneficial. In both female and male Dahl S rats, studies should focus on mechanisms of renal enlargement and worsening of proteinuria to determine if this is a pregnancy-specific effect.

## 5. Conclusions

This is the first study to test STS in a spontaneous rat model of preeclampsia and fetal growth restriction. Daily administration at 3 g per kg body weight reduced maternal blood pressure, holding promise for prolongation of preeclamptic pregnancies, and thus fetal outcomes. However, we also found deleterious effects, including worsening of proteinuria and placental insufficiency. Hence, STS cannot be recommended for clinical use during pregnancy at this stage.

## Figures and Tables

**Table 1:** Renal function parameters at the end of pregnancy.

Parameter	CON (n = 13)	STS (2 g) (n = 9)	STS (3 g) (n = 8)	*p*-Value
Water intake (mL) GD19	42.7 ± 1.9	48.0 ± 3.2	42.3 ± 3.6	0.30
Urine production GD19 (mL/kg BW per hour)	2.20 ± 0.2	2.21 ± 0.3	1.89 ± 0.1	0.59
Proteinuria GD19 (mg/24h)	56 ± 5 ^a^	51 ± 8	89 ± 15 ^a^	0.02
Urea plasma GD20 (mmol/L)	7.7 ± 0.2	8.5 ± 0.6	8.4 ± 0.6	0.57
Body weight (grams) GD19	306 ± 4	316 ± 7	294 ± 3	0.02
Gestational weight gain GD0-19 (%)	32.5 ± 1.9	37.9 ± 1.7	24.3 ± 1.6	<0.001

STS dose in grams STS per kg body weight per day. The *p*-value from one-way ANOVA is provided with similar superscripted letters indicating which treatment groups was significantly different from the control group in Dunnett’s multiple comparisons test. Data presented as mean ± S.E.M. CON, control; GD, gestational day; and STS, sodium thiosulfate.

**Table 2:** Effect of STS treatment on fetal and placental outcomes.

Fetal and Placental Outcome	CON (n = 13)	STS (2 g) (n = 9)	STS (3 g) (n = 8)	*p*-Value
Fetal weight (g)	2.13 ± 0.04	2.13 ± 0.07	2.01 ± 0.05	0.28
Fetal crown-rump length (cm)	3.06 ± 0.04	3.03 ± 0.05	3.11 ± 0.03	0.51
Viable litter size (n)	10.71 ± 0.6	11.2 ± 0.5	11.3 ± 0.4	0.68
Fetal resorptions (%)	8.4 ± 1.9	5.9 ± 2.4	3.0 ± 1.5	0.24
Placental weight (g)	0.50 ± 0.01	0.51 ± 0.01	0.53 ± 0.01	0.14
Fetal/placental weight ratio	4.31 ± 0.08 ^a^	4.27 ± 0.16	3.83 ± 0.07 ^a^	<0.01

STS dose in grams STS per kg body weight per day. The *p*-value from one-way ANOVA is provided with similar superscripted letters indicating which treatment groups was significantly different from the control group in Dunnett’s multiple comparisons test. Data presented as mean ± S.E.M. CON, control; STS, sodium thiosulfate.

**Table 3:** Organ weight and hematocrit on GD20.

Parameter	CON (n = 13)	2 g STS (n = 9)	3 g STS (n = 8)	*p*-Value
Relative total kidney weight (%)	0.72 ± 0.02 ^a^	0.75 ± 0.03	0.86 ± 0.04 ^a^	<0.01
Relative heart weight (%)	0.34 ± 0.004	0.35 ± 0.01	0.33 ± 0.01	0.28
Relative splenic weight (%)	0.28 ± 0.01	0.30 ± 0.01	0.31 ± 0.01	0.07
Hematocrit (%)	36.5 ± 1.7	38.4 ± 0.9	33.4 ± 2.1	0.18

STS dose in grams STS per kg body weight per day. The *p*-value from one-way ANOVA is provided with similar superscripted letters indicating which treatment groups was significantly different from the control group in Dunnett’s multiple comparisons test. Data presented as mean ± S.E.M. CON, control; GD, gestational day; STS, sodium thiosulfate.

**Table 4:** Spearman’s rho correlation between thiosulfate and sulfate and mean arterial pressure.

Sample (n = 30)	*r*	*p*-Value
Plasma thiosulfate (uM)	−0.62	<0.001
Plasma sulfate concentration (uM)	−0.26	0.17
Urinary thiosulfate excretion (umol/24 h)	−0.43	0.02
Urinary sulfate excretion (umol/24 h)	−0.45	0.01

**Table 5:** Histology scoring of kidney injury.

Kidney Injury Scoring Parameter	CON (n = 13)	STS 2 g (n = 9)	STS 3 g (n = 7)	*p*-value
Glomerulosclerosis damage score (%)	15.2 ± 2.5	10.2 ± 3.3	14.0 ± 5.8	0.58
Glomerular area (µm^2^)	10,906 ± 421	11,443 ± 516	10,279 ± 410	0.29
Glomerular tuft (µm^2^)	8944 ± 355	9390 ± 439	8502 ± 364	0.37
Urinary space (µm^2^)	1962 ± 84	2053 ± 129	1777 ± 155	0.31

STS dose in grams STS per kg body weight per day. Histological evaluation of kidneys showed that glomerulosclerosis in the control pregnant Dahl S rat (n = 13) was neither ameliorated nor exacerbated by either 2 g (n = 9) or 3 g (n = 7) STS per kg body weight per day. Data presented as mean ± S.E.M. CON, control; STS, sodium thiosulfate.

**Table 6:** Histology scoring of the placenta.

Placental Histology Scoring Parameter	CON (n = 12)	STS 2 g (n = 8)	STS 3 g (n = 8)	*p*-Value
Labyrinth zone (mm^2^)	21.89 ± 0.74	20.60 ± 0.95	21.64 ± 1.45	0.65
Basal zone (mm^2^)	7.61 ± 0.44	6.9 ± 0.85	8.67 ± 0.57	0.17
Proportion labyrinth zone of whole fetal placental area (%)	74 ± 1	76 ± 2	71 ± 1	0.22
Proportion basal zone of whole fetal placental area (%)	26 ± 1	25 ± 2	29 ± 1	0.22

STS dose in grams STS per kg body weight per day. Morphological evaluation of placentas showed no difference in the control pregnant Dahl S rat as compared with either 2 g or 3 g STS per kg body weight per day. Data presented as mean ± S.E.M. CON, control; STS, sodium thiosulfate.

## Appendix A

Supplementary figures.
